# Determination of risk factors of postoperative pneumonia in elderly patients with hip fracture: What can we do?

**DOI:** 10.1371/journal.pone.0273350

**Published:** 2022-08-23

**Authors:** Yibing Yu, Peiwen Zheng

**Affiliations:** Department of Orthopedics, Wuhan Fourth Hospital, Wuhan Orthopedic Hospital, Puai Hospital, Tongji Medical College, Huazhong University of Science and Technology, Wuhan, China; Assiut University Faculty of Medicine, EGYPT

## Abstract

**Background:**

Postoperative pneumonia is a serious complication in elderly patients with hip fracture. It is necessary to identify the influencing factors of postoperative pneumonia in patients with hip fracture.

**Methods:**

Elderly patients with hip fractures admitted to a tertiary hospital in China from January 1, 2020 to August 31, 2021 were included. The characteristics of patients with and without postoperative pneumonia were evaluated and compared. Logistic multivariate regression analyses were conducted to assess the risk factors of postoperative pneumonia.

**Results:**

267 patients with hip fracture were included, the incidence of postoperative pneumonia in patients with hip fracture was 13.11%. There were significant differences in the age, diabetes mellitus, anemia, hypoalbuminemia, anesthesia method and duration of surgery between infection and no infection group, no significant differences in the gender, BMI, hypertension, hyperlipidemia, type of fracture, preoperative oxygen saturation, white blood cell count, platelet count, red blood cell count, creatinine, alanine aminotransferase, aspartate aminotransferase, estimated blood loss during surgery were detected between infection and no infection group. Logistic regression analysis showed that age≥70y (OR2.326, 95%CI1.248~3.129), diabetes mellitus (OR2.123, 95%CI1.021~3.551), anemia (OR3.199,95%CI1.943~5.024), hypoalbuminemia (OR2.377, 95%CI1.211~3.398), general anesthesia (OR1.947, 95%CI1.115~3.038), duration of surgery≥120min (OR1.621, 95%CI1.488~2.534) were the risk factors of postoperative pneumonia in elderly patients with hip fracture (all p<0.05). Escherichia Coli (33.33%), Klebsiella pneumoniae (28.57%), Staphylococcus aureus (21.43%) were the most common bacteria of pulmonary infection.

**Conclusion:**

There are many risk factors for postoperative pneumonia in elderly patients with hip fractures after surgery. In clinical practice, medical workers should take targeted interventions for those risk factors to reduce postoperative pneumonia.

## Introduction

With the accelerating process of aging, the medical problems of elderly patients have become one of the key concerns of people’s livelihood. Among them, the incidence of fracture diseases in the elderly is relatively high, which brings a greater medical burden to patients and the medical system [1]. Hip fracture is one of the most common fracture types in elderly patients, and its incidence also increases with age [2]. According to reports [3, 4], the mortality and disability rate of elderly patients with hip fracture is extremely high, and the death of hip fracture patients is closely associated with postoperative complications. Among them, pulmonary infection is one of the most common complications after hip fracture in the elderly, 18.31% patients with hip fracture can have postoperative pulmonary infection, which may be associated with the elder age, longer bed and hospital stay [5]. Once a postoperative pulmonary infection occurs, it will seriously affect the patient’s postoperative recovery, prolong the hospital stay, increase the cost of hospitalization, and even cause the death of the patient [6, 7]. Therefore, the prevention and treatment of postoperative pulmonary infection in patients with hip fractures is of great significance to the prognosis of patients.

The rate of new pulmonary infections after hip fracture is 8.12% to 19.05% [8, 9]. Once patients have pulmonary infections, the risk of death within 30 days after surgery is 8 times that of other patients without pulmonary infections [10]. It can be seen that for postoperative patients with hip fracture, timely and reasonable preventive interventions for postoperative pulmonary infections are needed to improve the prognosis of patients [11]. To ensure the rationality of the implementation of preventive interventions for pulmonary infections after hip fractures in the elderly, it is necessary to understand a series of risk factors that may be associated with pulmonary infections. At present, although there are several reports on the risk factors of postoperative pulmonary infection after hip fracture surgery, due to the limitations of various research subjects, regions and populations, a unified understanding has not been reached so far, further studies from different area and populations are needed. Based on this background, we retrospectively analyzed the clinical data of elderly hip fracture patients undergoing surgical treatment in hospital in recent years, aiming to analyze the risk factors that may be associated to the hospital-acquired pulmonary infections in elderly hip fracture patients after surgery, to provide reference for the clinical development of reasonable plan for the prevention and treatment of postoperative pulmonary infection after hip fracture.

## Methods

### Ethical consideration

In this study, all methods were performed in accordance with the relevant guidelines and regulations. Approval from the ethical committee of Wuhan Fourth Hospital (approval number: A10085c) had been obtained in this study, and all the included patients were well informed and written informed consents had been obtained from all the included patients.

### Patients

We retrospectively selected elderly patients with hip fractures admitted from January 1, 2020 to August 31, 2021 as the study patients. The inclusion criteria of this study were: (1) The hip fracture was confirmed by MRI or X-ray. (2) The patient received artificial hip replacement or internal fixation in our hospital. (3) The clinical data was complete. (4) Age> 60 years old. (5) The patients were informed and agreed to participate in this study. Exclusion criteria in this study were: (1) Patients with other infectious diseases diagnosed before surgery. (2) Patients with coronavirus 2019 (COVID-19) infection. (3) Those who had received surgical treatment for fractures of the same site in the past since the patients that underwent previous surgery at the same site had higher risk of postoperative complications including pulmonary infection [12]. (4) Patients who were not willing to participate in this study.

### Diagnostic criteria for pulmonary infection

We referred to the relevant diagnostic criteria for diagnosing the hospital-acquired pneumonia during the hospital stay of patients according to the "Guidelines for the Diagnosis and Treatment of Hospital-Acquired Pneumonia" of the Chinese Medical Association Respiratory Medicine Branch [13]. The criteria were: (1) After the operation, the patient’s original respiratory disease-related symptoms aggravated, or sputum coughing, thick sputum coughing. (2) The patient developed fever after the operation. (3) Physical examination results showed signs of pulmonary consolidation, and pulmonary rales could be heard on auscultation. (4) White blood cell count <4 ×10^9^/L or>10×10^9^/L. (5) The chest X-ray film showed patchy infiltrates or interstitial changes. After excluding patients with pulmonary cancer, tuberculosis, pulmonary embolism and other pulmonary diseases, in the above items, if the patient meets any of (1)~(4) and also meets item (5), the diagnosis can be confirmed for pulmonary infection.

### Bacteria identification

The sputum of elderly patients with pulmonary infection was collected. The specimen was collected as early morning sputum, and the influence of oral bacteria was removed after gargle. And we asked the patient to cough as much as possible to take deep sputum. The coughed sputum would be sent to the sterile container for inspection in time. Bacterial samples were collected solely from the postoperative pneumonia group. Bacterial culture of sputum samples was conducted in the laboratory of our hospital, the inspection instrument for identifying the bacteria was the automatic microbial identification and drug sensitivity analyzer XE100 (France BioMérieux).

### Data collection

The two authors retrospectively reviewed the original records of patients’ medical records and collected the following relevant patient information: gender, age, body mass index(BMI), hypertension, diabetes mellitus, hyperlipidemia, anemia, hypoalbuminemia, type of fracture, preoperative oxygen saturation, preoperative laboratory examination including white blood cell count, platelet count, red blood cell count, creatinine, alanine aminotransferase, aspartate aminotransferase, anesthesia method, duration of surgery, estimated blood loss during surgery.

### Statistical analysis

We used SPSS 24.0 statistical software for data analysis. The measurement data were expressed as mean ± standard deviation, and the t test was used for group comparison. And the count data was expressed as the cases and percentage, Chi-square test was used for group analysis. The variables with statistical differences in the univariate analysis were further included in the logistic multivariate regression analysis. p<0.05 indicated that the difference was statistically significant in this present study.

## Results

### The characteristics of patients

267 postoperative patients with hip fracture were included in this study finally, of whom 35 patients had been diagnosed as postoperative pulmonary infection, the incidence of postoperative pulmonary infection in patients with hip fracture was 13.11%. As presented in Table 1, there were significant differences in the age, diabetes mellitus, anemia, hypoalbuminemia, anesthesia method and duration of surgery between infection and no infection group (all p<0.05). No significant differences in the gender, BMI, hypertension, hyperlipidemia, type of fracture, preoperative oxygen saturation, white blood cell count, platelet count, red blood cell count, creatinine, alanine aminotransferase, aspartate aminotransferase, estimated blood loss during surgery were detected between infection and no infection group (all p>0.05).

**Table 1:** The characteristics of included patients.

Items	Infection group(n = 35)	No infection group(n = 232)	t/χ^2^	p
Male/female	20/15	133/99	1.185	0.079
Age(y)	74.15 ± 10.23	65.08 ± 9.15	1.503	0.041
BMI (kg/m^2^)	23.11±2.75	22.87 ± 3.01	4.223	0.086
Hypertension	25(71.43%)	155(66.81%)	1.216	0.058
Diabetes mellitus	11(31.43%)	35(15.09%)	1.224	0.035
Hyperlipidemia	9(25.71%)	60(25.86%)	1.912	0.101
Anemia	19(54.29%)	24(10.34%)	1.095	0.033
Hypoalbuminemia	10(28.57%)	25(10.78%)	1.623	0.014
Type of fracture			1.178	0.079
Intertrochanteric fracture	16(45.71%)	104(44.83%)		
Femoral neck fracture	19(54.29%)	128(55.17%)		
Preoperative oxygen saturation(%)	95.14±8.01	94.72±9.17	10.442	0.083
Preoperative laboratory examination				
White blood cell count (×10^9^ ·L^-1^)	9.12±1.18	8.82±1.03	1.775	0.065
Platelet count (×10^9^ ·L^-1^)	214.09±83.22	211.45±102.16	1.202	0.114
Red blood cell count(×10^9^ ·L^-1^)	4.03±2.74	4.06±1.56	1.089	0.091
Creatinine (μmol·L^-1^)	32.35 ± 12.61	32.28 ± 14.27	2.235	0.102
Alanine aminotransferase (U ·L^-1^)	18.12±10.04	18.11±11.52	3.167	0.068
Aspartate aminotransferase(U ·L^-1^)	19.55±9.32	19.66±13.32	2.044	0.203
Anesthesia method			2.372	0.021
General anesthesia	26(74.29%)	122(52.59%)		
Lumbar anesthesia	9(25.71%)	110(47.41%)		
Duration of surgery(min)	148.23 ± 61.90	110.91 ± 77.34	10.959	0.015
Estimated blood loss during surgery(ml)	261.98±44.24	262.43±40.99	42.088	0.079

### Risk factors of pulmonary infection in elderly patients with hip fracture

The variable assignments of multivariate logistic regression were presented in Table 2. As indicated in Table 3, logistic regression analysis showed that age≥70y (OR2.326, 95%CI1.248~3.129), diabetes mellitus (OR2.123, 95%CI1.021~3.551), anemia (OR3.199,95%CI1.943~5.024), hypoalbuminemia (OR2.377, 95%CI1.211~3.398), general anesthesia (OR1.947, 95%CI1.115~3.038), duration of surgery≥120min (OR1.621, 95%CI1.488~2.534) were the risk factors of postoperative pulmonary infection in elderly patients with hip fracture (all p<0.05).

**Table 2:** The variable assignments of multivariate logistic regression.

Factors	Variables	Assignment
Pulmonary infection	Y	Yes = 1, no = 2
Age(y)	X_1_	≥70 = 1, <70 = 2
Diabetes mellitus	X_2_	Yes = 1, no = 2
Anemia	X_3_	Yes = 1, no = 2
Hypoalbuminemia	X_4_	Yes = 1, no = 2
Anesthesia method	X_5_	General anesthesia = 1, lumbar anesthesia = 2
Duration of surgery(min)	X_6_	≥120 = 1, <120 = 2

**Table 3:** Logistic regression analysis on the risk factors of postoperative pulmonary infection in elderly patients with hip fracture.

Variables	β	S $\bar{\text{x}}$	OR	95%CI	p
Age≥70y	0.124	0.211	2.326	1.248~3.129	0.015
Diabetes mellitus	0.141	0.138	2.123	1.021~3.551	0.012
Anemia	0.133	0.104	3.199	1.943~5.024	0.039
Hypoalbuminemia	0.125	0.113	2.377	1.211~3.398	0.026
General anesthesia	0.109	0.188	1.947	1.115~3.038	0.009
Duration of surgery≥120min	0.112	0.106	1.621	1.488~2.534	0.018

### Pathogen distributions

As presented in Table 4, of the 35 cases of pulmonary infection, a total of 42 pathogens were detected. Escherichia Coli (33.33%), Klebsiella pneumoniae (28.57%), Staphylococcus aureus (21.43%) were the most common bacteria of pulmonary infection in patients with hip fracture.

**Table 4:** The pathogen distributions of postoperative pulmonary infection in patients with hip fracture (n = 42).

Pathogens	Cases	Percent
Gram-positive bacteria	12	28.57%
Staphylococcus aureus	9	21.43%
Streptococcus	2	4.76%
Enterococcus	1	2.38%
Gram-negative bacteria	29	69.05%
Escherichia Coli	14	33.33%
Klebsiella pneumoniae	12	28.57%
Acinetobacter baumannii	3	7.14%
Fungus	1	2.38%
Candida albicans	1	2.38%
In total	42	100%

## Discussions

The incidence of postoperative pulmonary infection in elderly patients with hip fractures after surgery differs in regions with different economic levels [14], but the overall incidence is still relatively high, which is worthy of attention. In addition, postoperative pulmonary infection will further increase the risk of death in such patients [15]. Studies [16, 17] have shown that pulmonary infection is an independent risk factor for death after hip fracture in the elderly. Previous study [18] has found that 14% of elderly hip fracture patients with pulmonary infections died within 30 days, while the case fatality rate of patients with non-pulmonary infections is only 1.7% within 30 days. Another study [19] has showed that the risk of death for patients with pulmonary infection after hip fracture is 7.36 times higher than that of patients without pulmonary infection after surgery. Therefore, the prevention of postoperative pulmonary infection should be strengthened clinically in order to reduce the complications and mortality. The results of this study have found that the incidence of postoperative pulmonary infection in patients with hip fracture is 13.11%, which is similar to previous reports [20, 21]. And we have found that age≥70y, diabetes mellitus, anemia, hypoalbuminemia, general anesthesia, duration of surgery≥120min were the risk factors of postoperative pulmonary infection in elderly patients with hip fracture, early intervention targeted on those influencing factors should be taken to reduce the development of postoperative pulmonary infections.

Regarding the influence of age on postoperative pulmonary infections in elderly patients with hip fractures, many studies [22, 23] have reached different conclusions. A study [24] has reviewed 1,429 patients undergoing hip fracture surgery, and the results have showed that the higher the age, the greater the probability of postoperative pulmonary infections. This may be because elderly patients have more underlying diseases and the body’s immune function is reduced, which is extremely easy under the invasion of pathogenic bacteria, combined with fracture trauma, surgical stress, and long-term bed rest caused a further decline in immunity, which induced pulmonary infection [25–27]. However, some studies [28–30] have pointed out that the occurrence of postoperative pulmonary infections should be attributed to the increase in the number of comorbidities that accompany age, rather than age itself. However, it is currently recognized that the incidence of hip fractures in elderly patients increases with age, and surgery is the current main treatment method. Therefore, surgery cannot be given up on the grounds of advanced age, and other controllable patients should be evaluated. For patients with more risk factors, we should adopt targeted interventions to reduce the risks of postoperative pulmonary infections.

Malnutrition has been proven to be one of the important risk factors for postoperative pulmonary infection in elderly patients with hip fracture. In addition to low BMI, serum albumin is also an important indicator of nutritional status [31]. Studies [32, 33] have pointed out that low serum albumin levels increase the risk of pneumonia, because wounds, fracture healing, and muscle strength recovery require a large amount of protein supplementation. When protein is insufficient, muscle strength and limb function decline, causing the prolonging duration of bed time [34]. Besides, some patients are in a state of malnutrition when they are admitted to the hospital, coupled with increased catabolism caused by trauma and surgery, and increased demand for nutrients, resulting in a further sharp decline in nutritional status, weakened immunity, and increased risk of postoperative pulmonary infection [35]. Therefore, elderly patients with hip fractures should be screened for nutrition before surgery, and nutritional support interventions should be implemented before surgery to reduce the risk of postoperative pulmonary infections due to malnutrition.

Diabetes is one of the risk factors for postoperative pulmonary infection in elderly patients with hip fracture. Some studies [36, 37] have found that patients with diabetes before surgery are prone to pulmonary infection after surgery, which is consistent with the results of this study. However, some studies [38, 39] have pointed out that diabetes is not associated with the occurrence of postoperative pulmonary infections in elderly patients with hip fractures. Study [40] has reported that when the patient’s blood glucose is >200 mg/dL, it will increase the incidence of pneumonia. Diabetes may be associated with postoperative pulmonary infections in elderly patients with hip fractures, but this relationship may only exists when blood sugar is high [41]. The reason may be that when the blood sugar of diabetic patients is too high, the plasma osmotic pressure will also increase, which inhibits the phagocytic ability of immune cells in the blood and is prone to infection [42, 43]. However, due to the small sample size of related studies, there may be biases in the results. Further researches are needed to confirm the role of diabetes in the future.

Studies [44, 45] have reported that the longer duration of surgery is an important risk factor for postoperative pulmonary infection in elderly patients with hip fractures, which is more consistent with the results of this study. The prolonged operation time may be caused by a variety of factors, such as severe osteoporosis and complex fracture morphology [46]. The prolongation of the operation time can lead to an increase in blood loss during the operation, and the prolongation of the patient’s intraoperative hypothermia time, resulting in a decrease in their resistance, thereby increasing the postoperative pulmonary infection [47]. In addition, during general anesthesia surgery, the cough reflex is suppressed, and prolonged surgery leads to rapid proliferation of lower respiratory tract bacteria, which may lead to the occurrence of pneumonia [48]. There are reports [49, 50] which says delay hip fracture surgery increases the risk factor of pneumonia. Therefore, medical staff should shorten the operation time as much as possible, and pay attention to monitoring the patient’s body temperature and keep warm during the operation.

At present, many scholars [51, 52] believe that general anesthesia is related to the occurrence of pulmonary infections after surgery. Because local anesthesia does not affect the patient’s spontaneous breathing, does not inhibit the protective cough reflex, and at the same time avoids tracheal intubation, thus reducing the risk of postoperative pulmonary infection. But the shortcoming of the study is that doctors are more inclined to adopt general anesthesia for patients with more severe illness, which may cause selection bias. In addition, it’s been reported [53] that the incidence of pulmonary infection after local anesthesia is lower than that of patients under general anesthesia. However, when subgroup analysis is performed, only in patients with femoral intertrochanteric fractures, the difference in the results of the study is statistically significant, while in the patients with femoral neck, there is no such trend among fracture patients. However, the specific reasons are not clear, and further studies are needed in the future. The data about pathogen detection in 34 cases happened after the surgery or before the surgery should be compared, yet most included patients did not undergo pathogen detection before surgery in this study. this study has found that the bacteria in pulmonary infections are mainly gram-negative bacteria. This result suggests that the detection of pathogenic bacteria should also be paid attention to in actual clinical treatment, and reasonable antimicrobial treatment should be selected in combination with patient drug sensitivity test to improve anti-infection effects.

## Conclusions

In summary, there are many risk factors for hospital-acquired pulmonary infection after hip fracture in the elderly, for patients with age≥70y, diabetes mellitus, anemia, hypoalbuminemia, general anesthesia, duration of surgery≥120min, they may have higher risks of postoperative pulmonary infection. Actively correct the patient’s anemia, treat comorbidities, and improve pulmonary function before surgery, and give patients targeted intervention after surgery are necessary for patients with hip fracture. For patients who have already developed pulmonary infections, bacterial culture and drug sensitivity testing should be carried out as soon as possible to ensure the rationality of the use of antibacterial drugs.

## Supporting information

S1 File (DOCX)

S2 File (PDF)

S1 Checklist STROBE statement—checklist of items that should be included in reports of observational studies. (DOCX)

## List of abbreviation

BMI: body mass index
COVID-19: coronavirus 2019

## References

[1] Yuan Y, Tian W, Deng X, Yue R, Ge X, Wu X, et al: Elderly patients with concurrent hip fracture and lower respiratory tract infection: the pathogens and prognosis over different bedridden periods. J Orthop Surg Res 2021, 16(1):246. doi: 10.1186/s13018-021-02399-1 33849586 PMC8042877

[2] Kebaetse M, Nkhwa S, Mogodi M, Masunge J, Gureja YP, Ramabu M, et al: Epidemiology of hip fracture in Botswana. Arch Osteoporos 2021, 16(1):24. doi: 10.1007/s11657-021-00885-x 33550503 PMC7867517

[3] Jianwen Z, Xiaowei W, Tiansheng S: One-year mortality risk and risk factors analysis after hip fracture in elderly patients. Journal of Practical Orthopedics 2020, 26(5):5–8.

[4] Xiaowei W, Tiansheng S, Zhi L: Analysis of postoperative mortality in elderly patients with hip fracture Chinese Journal of Bone and Joint Injury 2021, 36(10):3–6.

[5] Man L, Bei T, Aili W: Application of multidisciplinary co-management mode under the concept of enhanced recovery surgery in perioperative period of emergency elderly hip fracture Modern Clinical Nursing 2020, 19(5):6–10.

[6] Skuladottir SS, Ramel A, Hjaltadottir I, Launer LJ, Cotch MF, Siggeirsdottir K, et al: Characteristics of incidence hip fracture cases in older adults participating in the longitudinal AGES-Reykjavik study. Osteoporos Int 2021, 32(2):243–250. doi: 10.1007/s00198-020-05567-x 32808140 PMC11190885

[7] Makridis KG, Badras LS, Badras SL, Karachalios TS: Searching for the ’winner’ hip fracture patient: the effect of modifiable and non-modifiable factors on clinical outcomes following hip fracture surgery. Hip Int 2021, 31(1):115–124. doi: 10.1177/1120700019878814 31547719

[8] Chang SC, Lai JI, Lu MC, Lin KH, Wang WS, Lo SS, et al: Reduction in the incidence of pneumonia in elderly patients after hip fracture surgery: An inpatient pulmonary rehabilitation program. Medicine (Baltimore) 2018, 97(33):e11845. doi: 10.1097/MD.0000000000011845 30113476 PMC6113002

[9] Lo IL, Siu CW, Tse HF, Lau TW, Leung F, Wong M: Pre-operative pulmonary assessment for patients with hip fracture. Osteoporos Int 2010, 21(Suppl 4):S579–586. doi: 10.1007/s00198-010-1427-7 21057997 PMC2924432

[10] Wong JK, Kim TE, Mudumbai SC, Memtsoudis SG, Giori NJ, Howard SK, et al: Are Case Volume and Facility Complexity Level Associated With Postoperative Complications After Hip Fracture Surgery in the Veterans Affairs Healthcare System? Clin Orthop Relat Res 2019, 477(1):177–190. doi: 10.1097/CORR.0000000000000460 30179946 PMC6345301

[11] Goh EL, Lerner RG, Achten J, Parsons N, Griffin XL, Costa PML: Complications following hip fracture: Results from the World Hip Trauma Evaluation cohort study. Injury 2020, 51(6):1331–1336. doi: 10.1016/j.injury.2020.03.031 32268962 PMC7322551

[12] Wenqian Y, Dong Y: Risk factors for death 6 months after surgery in elderly patients with hip fracture. Journal of Trauma Surgery 2021, 23(1):3–6.

[13] Association RMBoCM: Guidelines for the diagnosis and treatment of hospital-acquired pneumonia and ventilator-associated pneumonia in Chinese adult hospitals (2018 edition). Chinese Journal of Tuberculosis and Respiratory Medicine 2018, 41(4):255–280.

[14] Ravi B, Pincus D, Khan H, Wasserstein D, Jenkinson R, Kreder HJ: Comparing Complications and Costs of Total Hip Arthroplasty and Hemiarthroplasty for Femoral Neck Fractures: A Propensity Score-Matched, Population-Based Study. J Bone Joint Surg Am 2019, 101(7):572–579. doi: 10.2106/JBJS.18.00539 30946190

[15] Chen J, Wang X, Qian H, Ye J, Qian J, Hua J: Correlation between common postoperative complications of prolonged bed rest and quality of life in hospitalized elderly hip fracture patients. Ann Palliat Med 2020, 9(3):1125–1133. doi: 10.21037/apm-20-891 32498527

[16] Patterson JT, Bohl DD, Basques BA, Arzeno AH, Grauer JN: Does Preoperative Pneumonia Affect Complications of Geriatric Hip Fracture Surgery? Am J Orthop (Belle Mead NJ) 2017, 46(3):E177–E185. 28666049

[17] Gallardo-Calero I, Larrainzar-Coghen T, Rodriguez-Pardo D, Pigrau C, Sanchez-Raya J, Amat C, et al: Increased infection risk after hip hemiarthroplasty in institutionalized patients with proximal femur fracture. Injury 2016, 47(4):872–876. doi: 10.1016/j.injury.2015.12.032 26857632

[18] Phillips CB, Barrett JA, Losina E, Mahomed NN, Lingard EA, Guadagnoli E, et al: Incidence rates of dislocation, pulmonary embolism, and deep infection during the first six months after elective total hip replacement. J Bone Joint Surg Am 2003, 85(1):20–26. doi: 10.2106/00004623-200301000-00004 12533567

[19] Macey ARM, Butler J, Martin SC, Tan TY, Leach WJ, Jamal B: 30-day outcomes in hip fracture patients during the COVID-19 pandemic compared to the preceding year. Bone Jt Open 2020, 1(7):415–419. doi: 10.1302/2633-1462.17.BJO-2020-0077.R1 33215132 PMC7659666

[20] Agrawal S, Turk R, Burton BN, Ingrande J, Gabriel RA: The association of preoperative delirium with postoperative outcomes following hip surgery in the elderly. J Clin Anesth 2020, 60:28–33. doi: 10.1016/j.jclinane.2019.08.015 31437598

[21] Mi B, Chen L, Tong D, Panayi AC, Ji F, Guo J, et al: Delayed surgery versus nonoperative treatment for hip fractures in post-COVID-19 arena: a retrospective study of 145 patients. Acta Orthop 2020, 91(6):639–643. doi: 10.1080/17453674.2020.1816617 32896189 PMC8023940

[22] Morgan L, McKeever TM, Nightingale J, Deakin DE, Moppett IK: Spinal or general anaesthesia for surgical repair of hip fracture and subsequent risk of mortality and morbidity: a database analysis using propensity score-matching. Anaesthesia 2020, 75(9):1173–1179. doi: 10.1111/anae.15042 32337715

[23] Sasabuchi Y, Matsui H, Lefor AK, Fushimi K, Yasunaga H: Timing of surgery for hip fractures in the elderly: A retrospective cohort study. Injury 2018, 49(10):1848–1854. doi: 10.1016/j.injury.2018.07.026 30097309

[24] Lv H, Yin P, Long A, Gao Y, Zhao Z, Li J, et al: Clinical characteristics and risk factors of postoperative pneumonia after hip fracture surgery: a prospective cohort study. Osteoporos Int 2016, 27(10):3001–3009. doi: 10.1007/s00198-016-3624-5 27241669

[25] Wang X, Dai L, Zhang Y, Lv Y: Gender and Low Albumin and Oxygen Levels are Risk Factors for Perioperative Pneumonia in Geriatric Hip Fracture Patients. Clin Interv Aging 2020, 15:419–424. doi: 10.2147/CIA.S241592 32256056 PMC7090213

[26] Folbert EC, Hegeman JH, Gierveld R, van Netten JJ, Velde DV, Ten Duis HJ, et al: Complications during hospitalization and risk factors in elderly patients with hip fracture following integrated orthogeriatric treatment. Arch Orthop Trauma Surg 2017, 137(4):507–515. doi: 10.1007/s00402-017-2646-6 28233062

[27] Wang Y, Li X, Ji Y, Tian H, Liang X, Li N, et al: Preoperative Serum Albumin Level As A Predictor Of Postoperative Pneumonia After Femoral Neck Fracture Surgery In A Geriatric Population. Clin Interv Aging 2019, 14:2007–2016. doi: 10.2147/CIA.S231736 32009780 PMC6859085

[28] Dallari D, Zagra L, Cimatti P, Guindani N, D’Apolito R, Bove F, et al: Early mortality in hip fracture patients admitted during first wave of the COVID-19 pandemic in Northern Italy: a multicentre study. J Orthop Traumatol 2021, 22(1):15–19. doi: 10.1186/s10195-021-00577-9 33818650 PMC8020826

[29] Wiedl A, Forch S, Fenwick A, Mayr E: Incidence, Risk-Factors and Associated Mortality of Complications in Orthogeriatric Co-Managed Inpatients. Geriatr Orthop Surg Rehabil 2021, 12(5):215–226. doi: 10.1177/2151459321998314 33786204 PMC7961710

[30] Chen Z, Wu H, Jiang J, Xu K, Gao S, Chen L, et al: Nutritional risk screening score as an independent predictor of nonventilator hospital-acquired pneumonia: a cohort study of 67,280 patients. BMC Infect Dis 2021, 21(1):313–320. doi: 10.1186/s12879-021-06014-w 33794788 PMC8013169

[31] Scott BL, King CA, Lee CS, Lee MJ, Su EP, Landy DC: Periprosthetic Hip Fractures Outside the Initial Postoperative Period: Does Time from Diagnosis to Surgery Matter? Arthroplast Today 2020, 6(3):628–633. doi: 10.1016/j.artd.2020.06.008 32995412 PMC7502573

[32] Moller AM, Villebro N, Pedersen T, Tonnesen H: Effect of preoperative smoking intervention on postoperative complications: a randomised clinical trial. Lancet 2002, 359(9301):114–117. doi: 10.1016/S0140-6736(02)07369-5 11809253

[33] Dalton A, Zafirova Z: Preoperative Management of the Geriatric Patient: Frailty and Cognitive Impairment Assessment. Anesthesiol Clin 2018, 36(4):599–614. doi: 10.1016/j.anclin.2018.07.008 30390781

[34] Pino JE, Shah V, Ramos Tuarez FJ, Kreidieh OI, Donath E, Lovitz LS, et al: The utility of pulmonary function testing in the preoperative risk stratification of patients undergoing transcatheter aortic valve replacement. Catheter Cardiovasc Interv 2020, 95(6):179–185. doi: 10.1002/ccd.28402 31313472

[35] Savvidis C, Tournis S, Dede AD: Obesity and bone metabolism. Hormones (Athens) 2018, 17(2):205–217. doi: 10.1007/s42000-018-0018-4 29858847

[36] Formiga F, Freitez Ferreira MD, Montero A: [Diabetes mellitus and risk of hip fracture. A systematic review]. Rev Esp Geriatr Gerontol 2020, 55(1):34–41. 31668564 10.1016/j.regg.2019.08.009

[37] Guzon-Illescas O, Perez Fernandez E, Crespi Villarias N, Quiros Donate FJ, Pena M, Alonso-Blas C, et al: Mortality after osteoporotic hip fracture: incidence, trends, and associated factors. J Orthop Surg Res 2019, 14(1):203–212. doi: 10.1186/s13018-019-1226-6 31272470 PMC6610901

[38] Lei W, Gengsheng W, Congxiao Z, Mengzi X: Analysis of risk factors for postoperative pulmonary complications of elderly patients with hip fracture. Chinese Journal of Clinicians 2020, 48(11):81–83.

[39] Linhua H: pathogenic bacteria and related factors of elderly hip fracture complicated with lung infection. Chinese Journal of Coal Industry Medicine 2019, 22(3):53–56.

[40] Xiaobin C, Jianzheng Z, Jianwen Z: Analysis of death risk and risk factors in elderly patients with hip fracture within 30 days after operation. Chinese Journal of Geriatrics 2020, 39(7):813–816.

[41] Jianwen Z, Xiaowei W, Tiansheng S: Analysis of the risk of death and risk factors in elderly patients with hip fracture after one year. Journal of Practical Orthopedics 2020, 26(5):19–23.

[42] Hsu JY, Cheng CY, Hsu CY: Type 2 diabetes mellitus severity correlates with risk of hip fracture in patients with osteoporosis. Neth J Med 2018, 76(2):65–71. 29515003

[43] Mohsin S, Kaimala S, Sunny JJ, Adeghate E, Brown EM: Type 2 Diabetes Mellitus Increases the Risk to Hip Fracture in Postmenopausal Osteoporosis by Deteriorating the Trabecular Bone Microarchitecture and Bone Mass. J Diabetes Res 2019, 12(3):10–19. doi: 10.1155/2019/3876957 31815147 PMC6878775

[44] Xiaowei W, Tiansheng S, Zhi L: risk factors and prognosis of pulmonary infection after hip fracture in the elderly. Chinese Journal of Bone and Joint 2020, 9(9):645–649.

[45] Peng Z, Xiaolei S, Bingchen S: The clinical significance of the scoring system of the elderly operation risk scoring system in Daping Orthopedics for the implementation of stratified treatment of elderly patients with hip fractures after operation. Chinese Journal of Trauma 2020, 36(1):45–50.

[46] Shaoguang L, Zhi L, Tiansheng S: Analysis of 1-year mortality and risk factors in elderly hip fractures after operation. Beijing Medicine 2015, 37(11):1031–1035.

[47] Malik AT, Quatman CE, Phieffer LS, Ly TV, Khan SN: Timing of complications following surgery for geriatric hip fractures. J Clin Orthop Trauma 2019, 10(5):904–911. doi: 10.1016/j.jcot.2018.10.020 31528066 PMC6739463

[48] Malik AT, Quatman CE, Phieffer LS, Jain N, Khan SN, Ly TV: 30-day adverse events, length of stay and re-admissions following surgical management of pelvic/acetabular fractures. J Clin Orthop Trauma 2019, 10(5):890–895. doi: 10.1016/j.jcot.2019.02.010 31528063 PMC6739240

[49] Xujuan C, Xiaobing Y, Rongmin Q: Research progress on risk factors of postoperative pulmonary infection in elderly patients with hip fracture. Journal of Clinical and Pathology 2020, 40(7):8–11.

[50] Zeming Z, Shi X, Hejie W: Effect of surgical timing on the prognosis of elderly hip fractures. China Medical Herald 2021, 18(9):5–8.

[51] Gupta RM, Parvizi J, Hanssen AD, Gay PC: Postoperative complications in patients with obstructive sleep apnea syndrome undergoing hip or knee replacement: a case-control study. Mayo Clin Proc 2001, 76(9):897–905. doi: 10.4065/76.9.897 11560300

[52] Byun SE, Shon HC, Kim JW, Kim HK, Sim Y: Risk factors and prognostic implications of aspiration pneumonia in older hip fracture patients: A multicenter retrospective analysis. Geriatr Gerontol Int 2019, 19(2):119–123. doi: 10.1111/ggi.13559 30556343

[53] Kuza CM, Hatzakis G, Nahmias JT: The Assignment of American Society of Anesthesiologists Physical Status Classification for Adult Polytrauma Patients: Results From a Survey and Future Considerations. Anesth Analg 2017, 125(6):1960–1966. doi: 10.1213/ANE.0000000000002450 28891913

