# Supplementary material for: Determination of risk factors of postoperative pneumonia in elderly patients with hip fracture: What can we do?
Source: PLoS One. 2022 Aug 23;17(8):e0273350. doi: 10.1371/journal.pone.0273350 (PMC9398012; doi:10.1371/journal.pone.0273350)
Supplement: S1 File — (DOCX) [file pone.0273350.s001.docx]

**武汉市第四人民医院医学伦理委员会**

ICE of Wuhan Fourth Hospital

审查批件

Approval notice

Approval number: A10085c

| 研究方案  Protocol title | 老年髋部骨折患者术后并发肺炎的影响因素分析Analysis of the influencing factors of postoperative pneumonia in elderly patients with hip fracture | |
| --- | --- | --- |
| 任务来源  Source | 自主研究 Self research | |
| 课题编号  Protocol number | NA | |
| 研究单位和主要负责人  Study setting and principal investigator | 郑佩文 Zheng Peiwen | |
| 审查方式/类别  Type of review | ■快速审查 Quick review | |
| 会议时间  Meeting Date | 2019.12.16 | |
| 审阅文件  EC Submission Documents | 初审  文件Preliminary review document | 1、伦理审查申请表Ethical Review Application Form |
|  |  | 2、研究方案 Research plan |
|  |  | 3、受试者知情同意书 Subject's informed consent |
| 出席人数  Attendance | 全体委员20人，出席15人，缺席5人There were 20 members, 15 were present and 5 were absent | |
| 表决情况  Voting | 同意15人，作必要修改后同意0人，不同意0人，终止或暂停先前批准的试验0 人，回避0人  15 people agree, 0 people agree after making necessary modifications, 0 people disagree, 0 people terminate or suspend previously approved trials, 0 people avoid | |
| 审杳意见Evaluation comments: “同意”。”Agreed” | | |
| 意见说明Comments description:  在研究进行过程中研究者应于每年12月底向本伦理委员会汇报研究进展情况，研究负责人必须严格使用经过审查的相关研究方案。如果己到达伦理审査批件规定的有效时间，尚不能完成所有的研究工作，应在批件失效前一个月，递交持续审査申请；如研究结朿且在批件有效期内，需填写并上报任何不良事件（SAE)，均应立刻报告本伦理委员会。 所有研究方案的修改必须递交研究方案修改申请表，后经伦理委员会审査批准后方能执行。During the research process, the researcher should report the research progress to this ethics committee at the end of December each year, and the research leader must strictly use the relevant research plan that has been reviewed. If it has reached the effective time specified in the ethics review approval document and cannot complete all the research work, an application for continuous review should be submitted one month before the approval document expires; if the research is completed and within the validity period of the approval document, any defects must be filled in and reported All incidents (SAE) should be reported to this ethics committee immediately. All the modification of the research protocol must be submitted to the research protocol modification application form, and then can be implemented after the review and approval of the ethics committee. | | |
| 伦理审查批件有效期Validity Period of Ethics Review Approval | 2020年1月1日至2021年12月31日January 1, 2020 to December 31, 2021 | |
| *依据国家相关法规，本伦理委员会的组织和实施相对独立。  *According to relevant national regulations, the organization and implementation of this ethics committee is relatively independent.  *本伦理委员会的人员组成和工作程序是国家相关规定的，符合《赫尔辛基宣言》  *The personnel composition and working procedures of this ethics committee are in accordance with relevant national regulations and comply with the "Declaration of Helsinki"  武汉市第四人民医院医学伦理委员会  Ethics Committee of Wuhan Fourth People's Hospital | | |
