# Supplementary material for: Determination of risk factors of postoperative pneumonia in elderly patients with hip fracture: What can we do?
Source: PLoS One. 2022 Aug 23;17(8):e0273350. doi: 10.1371/journal.pone.0273350 (PMC9398012; doi:10.1371/journal.pone.0273350)
Supplement: S2 File — (PDF) [file pone.0273350.s002.pdf]

武汉市第四人民医院医学伦理委员会

ICE of Wuhan Fourth Hospital

审查批件

Approval notice

批件号/Approval number: A10085c

研究方案<br>Protocol title	老年髋部骨折患者术后并发肺炎的影响因素分析	
任务来源<br>Source	自主研究	
课题编号<br>Protocol number	NA	
研究单位和主要负责人<br>Study setting and principal investigator	郑佩文	
审查方式/类别<br>Type of review	口会议审查 ■快速审查	
会议时间<br>Meeting Date	2019.12.16	
审阅文件<br>EC Submission Documents	初审<br>文件	1、伦理审查申请表<br>2、研究方案<br>3、受试者知情同意书
出席人数<br>Attendance	全体委员 20 人，出席 15 人，缺席 5 人	
表决情况<br>Voting	同意 15 人，作必要修改后同意 0 人，不同意 0 人，终止或暂停先前批准的试验 0 人，回避 0 人	
审查意见 Evaluation comments:审查结果为“同意”。		
意见说明 Comments description:<br>在研究进行过程中研究者应于每年 12 月底向本伦理委员会汇报研究进展情况，研究负责人必须严格使用经过审查的相关研究方案。如果已到达伦理审查批件规定的有效时间，尚不能完成所有的研究工作，应在批件失效前一个月，递交持续审查申请；如研究结束且在批件有效期内，需填写并上报任何不良事件（SAE)，均应立刻报告本伦理委员会。 所有研究方案的修改必须递交研究方案修改申请表，后经伦理委员会审查批准后方能执行。		
伦理审查批件有效期	2020 年 1 月 1 日至 2021 年 12 月 31 日	

*依据国家相关法规，本伦理委员会的组织和实施相对独立。

*本伦理委员会的人员组成和工作程序是国家相关规定的，符合《赫尔辛基宣言》

武汉市第四人民医院伦理委员会

武汉市第四人民医院伦理委员会
